# Exploring solar dynamo behavior using an annually resolved carbon-14 compilation during multiple grand solar minima

**DOI:** 10.1038/s41598-024-55317-w

**Published:** 2024-03-07

**Authors:** Fadil Inceoglu

**Affiliations:** 1 Cooperative Institute for Research in Environmental Sciences, University of Colorado Boulder, Boulder, CO 80309 USA; 2 National Centers for Environmental Information, National Oceanic and Atmospheric Administration, Boulder, CO 80309 USA

**Keywords:** Sun, Solar dynamo, Grand solar minima, Carbon-14, Solar activity, Solar physics, Solar physics, Solar physics, Solar physics

## Abstract

In this study, we, for the first time, compiled all publicly available annually or biannually resolved ^14^C records, which fully covers 7 grand solar minima at an annual and bi-annual resolution. Our results from 7 grand solar minima showed a clear relationship between the rate of decrease (increase) in solar activity levels and how long the onset (termination) of the grand solar minima will last. Additionally, we show a weaker relationship between the durations of onsets and terminations and between the rate of increase and decrease of solar activity levels. Our results also suggest there might be two or more types of grand solar minima.

## Introduction

The Sun governs the conditions in the heliosphere through its magnetic activity with timescales ranging from minutes to multiple decades, creating space weather and space climate conditions. Observations of the Sun since the beginning of the 1600s have revealed that the Sun shows darker spots on its surface, so-called sunspots, and their total numbers on the solar disk varies cyclically with a period ranging from 9 to 13 years, which is also known as the Schwabe or Solar Cycle^1^. The number of sunspots on the solar surface is directly linked to the underlying magnetic activity cycle of the Sun, the Hale cycle^2^. However it is known that there is a lower threshold value of $\sim$ 1500 G for a sunspot to emerge^3^, meaning that even though the Sun continues its magnetic activity cycle, if the magnetic field is not strong enough, sunspots might not form, while active regions with weaker magnetic fields will continue to persist.

Information about the variations in past solar activity before the sunspot observations relies on the past production rates of cosmogenic radionuclides, which are produced in Earth’s atmosphere by interactions of Galactic Cosmic Rays (GCRs) from space with atmospheric species such as N and O^4^. Their production rates are inversely correlated with the solar and geomagnetic field strengths as a result of the nonlinear interactions between the Solar and terrestrial magnetic fields, and GCRs, meaning that a stronger solar magnetic field will result in lower cosmogenic radionuclide production rates^4^. The most well known and widely used cosmogenic radionuclides are ^10^Be in ice cores and ^14^C in tree rings for studies concerning the past solar activity^5–15^.

The Schwabe Cycles are modulated on longer time-scales as exhibited by grand solar minima, such as the Maunder Minimum (1645–1715), and with grand maxima, such as the Medieval Maximum (1100–1250)^16^, analogues of which were observed over the last $\sim$ 10,000 years^10,12,15,17^.

The persistence of the Schwabe cycles during grand solar minima is still an open question as annually and/or biannually resolved past production records of the ^14^C record only extends continuously back to 950 AD^13^, while the longest annually-resolved continuous ^10^Be record extends back only to 1389 AD^8^. It should be noted that there are some annually/bi-annually resolved ^14^C measurements before $\sim$ 950 AD, however there are gaps spanning a few hundreds to thousands of years in between them. In addition, as their geochemical behaviors in the Earth system allow for the high-frequency effects of the regional weather and climatic conditions to interfere, using ^10^Be as a proxy for the past solar activity levels poses a challenge, especially when the timescales under consideration is sub-decadal^9^.

Previous studies suggested that the cyclic behavior of solar magnetic activity does not cease during grand minimum states^7,8,10,13,18–22^. However, using annual ^14^C measurements from Danish oak, it was shown that ^14^C measurements did not have statistically significant signals associated with the Schwabe Cycle during the Spörer and Maunder Minima^23^. Further, following the termination of these two minima, a periodicity of $\sim$ 8 year was observed, suggesting a preliminary recovery of the Schwabe Cycles^23^. Another study suggested that the length of the Schwabe cycles before the onset of the Maunder Minimum increased significantly^24^. This was supported by a more recent study, which also suggested that a similar lengthening behavior can be observed prior to the Spörer Minimum^14^.

The difficulty in studying the changes in lengths and amplitudes of the Schwabe cycles before, during, and after grand solar minima arises from not having annually and/or biannually resolved continuous data, covering several grand solar minima. Even though there are currently publicly available annually and/or biannually resolved ^14^C records^13,14,24–26^, these records only extend continuously back to around 950 AD, while a longer ^14^C record is available with varying resolutions, most part of which is 5 years, the so-called IntCal20^27^. This is a compilation of various ^14^C records from all over the world extending back to 55000 BP (before present, before 1950). The first 13,900 years of the IntCal20 curve is based on tree rings as a fully atmospheric record^27^.

In this study, we for the first time, compiled all publicly available annual and bi-annual ^14^C records, fully covering 7 grand solar minima. Based on this compilation, we studied the behavior of solar dynamo during the onsets and terminations of these minima, including the relationship between the speed in which the Sun goes into and gets our of a solar minimum as well as the cycle and amplitude changes during these periods.

## Results

**Figure 1:**
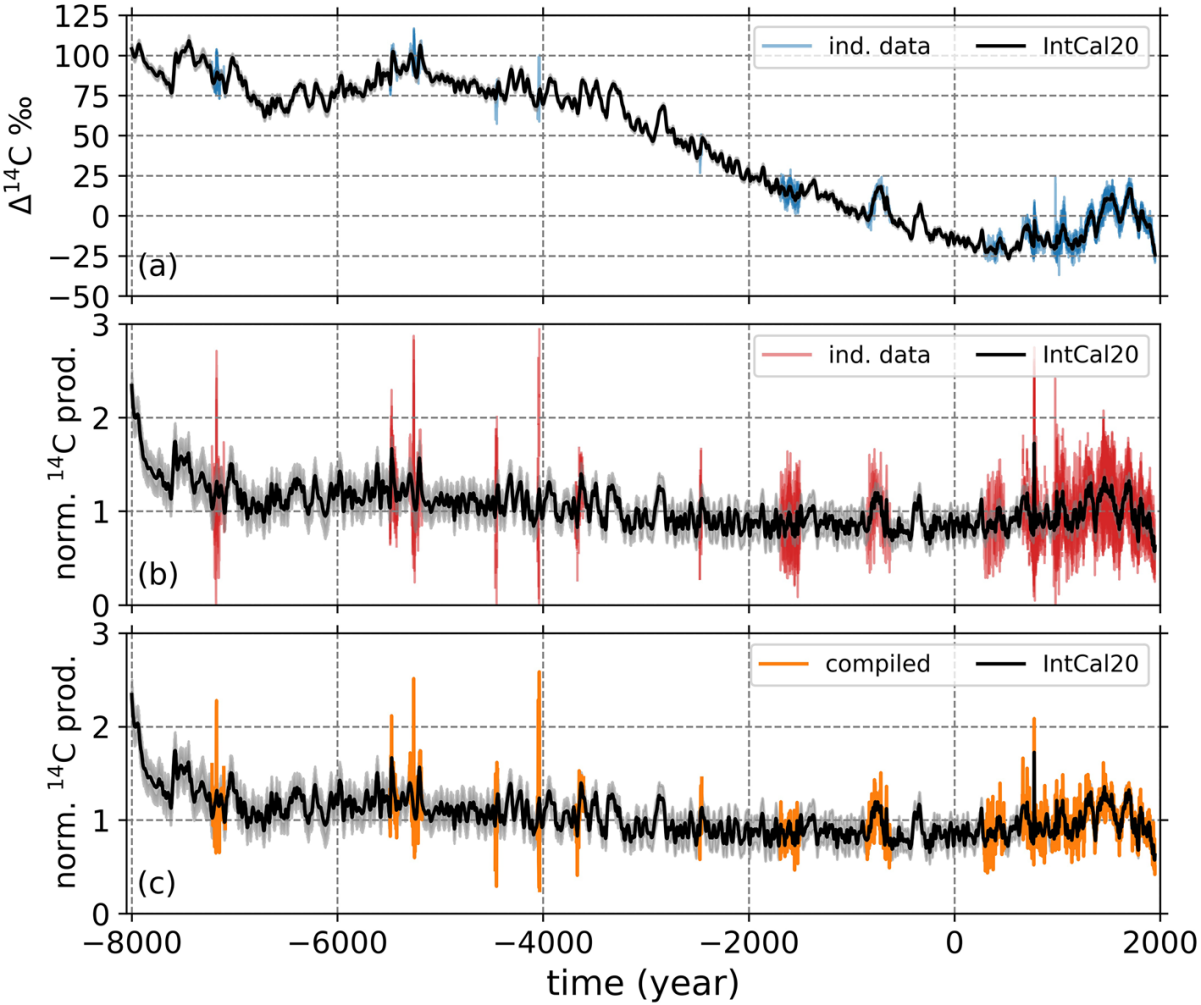
(**a**) Shows the IntCal20 curve (black) together with each individual ^14^C data used in our study (blue). The shaded areas show the standard deviations for each data point. (**b**) Shows the normalized ^14^C production rates calculated using a box-diffusion carbon model for the IntCal20 (black) and each individual ^14^C record (red) with their calculated standard deviations as black and red shades. (**c**) Shows the unfiltered IntCal20 (black) and the compilation (orange) ^14^C production rates with their standard deviation values (black and orange shades) The positive time axis shows calendar dates in AD, while the negative values indicate BC years.

The IntCal20 ^14^C curve and also each individual ^14^C record that are used in this study show a long-term decreasing trend through the past 10,000 years (the Holocene), while having periods of higher ^14^C values superimposed on it (Fig. 1a). The long-term trend observed in the ^14^C measurements results mainly from the variations in geomagnetic field intensities and the global carbon cycle^28^.

To remove the effects of the global carbon cycle from the *measured data* and to calculate the past production rates of ^14^C, which is only influenced by the solar magnetic activity and variations in geomagnetic field, we used a box-diffusion carbon model using IntCal20 and individual high-temporal resolution ^14^C records as inputs (see “Methods” section for details). The normalized ^14^C production rates through the Holocene does not exhibit the long-term decreasing trend as seen in the $\Delta ^{14}$C values (Fig. 1a), while still containing the long-term variations related to the changes in the geomagnetic field strength and the shorter-term higher and lower production rate periods, which are related to the solar activity levels (Fig. 1b).

Following that, we compiled all available individual production rates of ^14^C, most of which are used to generate the IntCal20 curve^27^. The compilation is made using the Bootstrapping method (see “Methods” section), which also allowed us to calculate the Gaussian noise that might be contained in each individual measurement and therefore the standard deviation for each complied data point (see “Methods” section, Fig. 1c). This led us to have two records of ^14^C production rates; a low-resolution record (LR) based on the IntCal20 and a high-resolution record (HR) compiled based on all individual ^14^C records. For further analyses, we only use the HR series.

**Figure 2:**
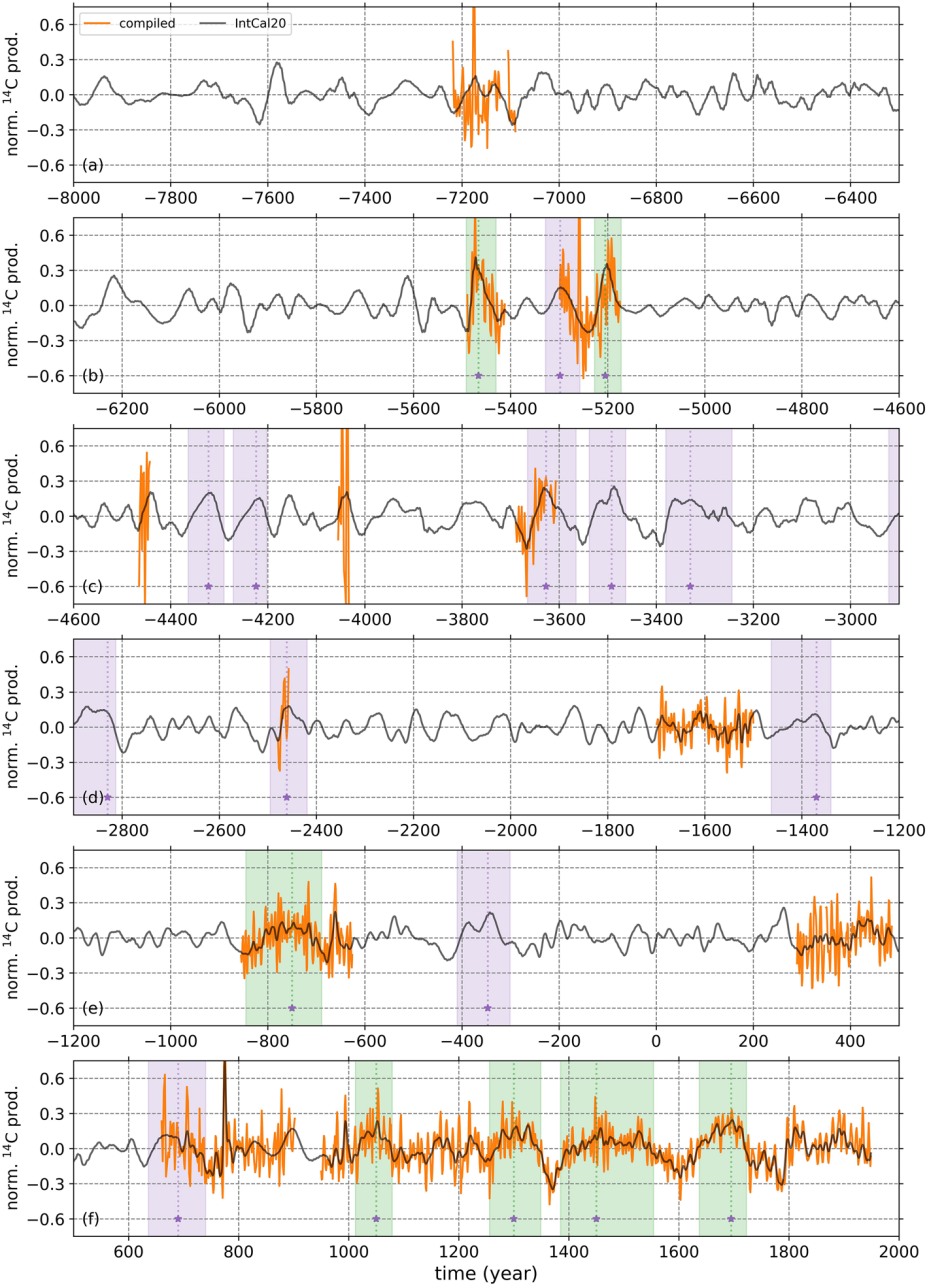
The past production rates of ^14^C from the IntCal20 (black) and our compilation (orange) during grand solar minima from 8000 BC to 1950 AD. The purple stars indicate the center of grand solar minima, while the purple shades show the full duration of grand solar minima previously identified in Refs.^10,12^. The green shades indicate where our HR compilation covers the full duration of a grand minimum.

Even though we removed the effects of the global carbon cycle, the HR and LR ^14^C production rate curves still bear effects not only from the solar activity, but also from the variations in the geomagnetic activity. It was suggested that the variations longer than multi-millennia timescales in the production rates are influenced by the geomagnetic field^29–33^. Therefore, to reduce the effects of the geomagnetic field on the past production rates of ^14^C, we high-pass filtered the data using a Butterworth filter of order 5 with cutoff frequencies at (1/250) year^-1^ (Fig. 2) (see “Methods” section). The study period, which extends from 1950 AD back to 8000 BC, covers 18 previously identified grand solar minima^10,17^, which can be observed as increases in the ^14^C production rates, as the production of cosmogenic radionuclides are inversely correlated with the solar activity levels. One can observe that 7 of these grand solar minima are fully covered by our compilation, while currently there are no high-resolution ^14^C measurements during the remaining 7 grand solar minimum periods. Additionally, there are 3 grand minima where there are available ^14^C data partially covering these periods (Fig. 2a–f). For our further analyses, we only considered the 7 grand solar minima, where we have the full ^14^C production rate coverage. We must note that the center dates of the identified grand solar minima are taken from Refs.^10,12^, where authors used zero-crossing method to detect the onset and termination dates, and the highest amplitude point to detect the center date of these grand solar minima. In our study, we defined duration of the onset and termination as the time period which lasts from the start of the grand minima until the peak date and from the peak date to its termination date, respectively.

**Figure 3:**
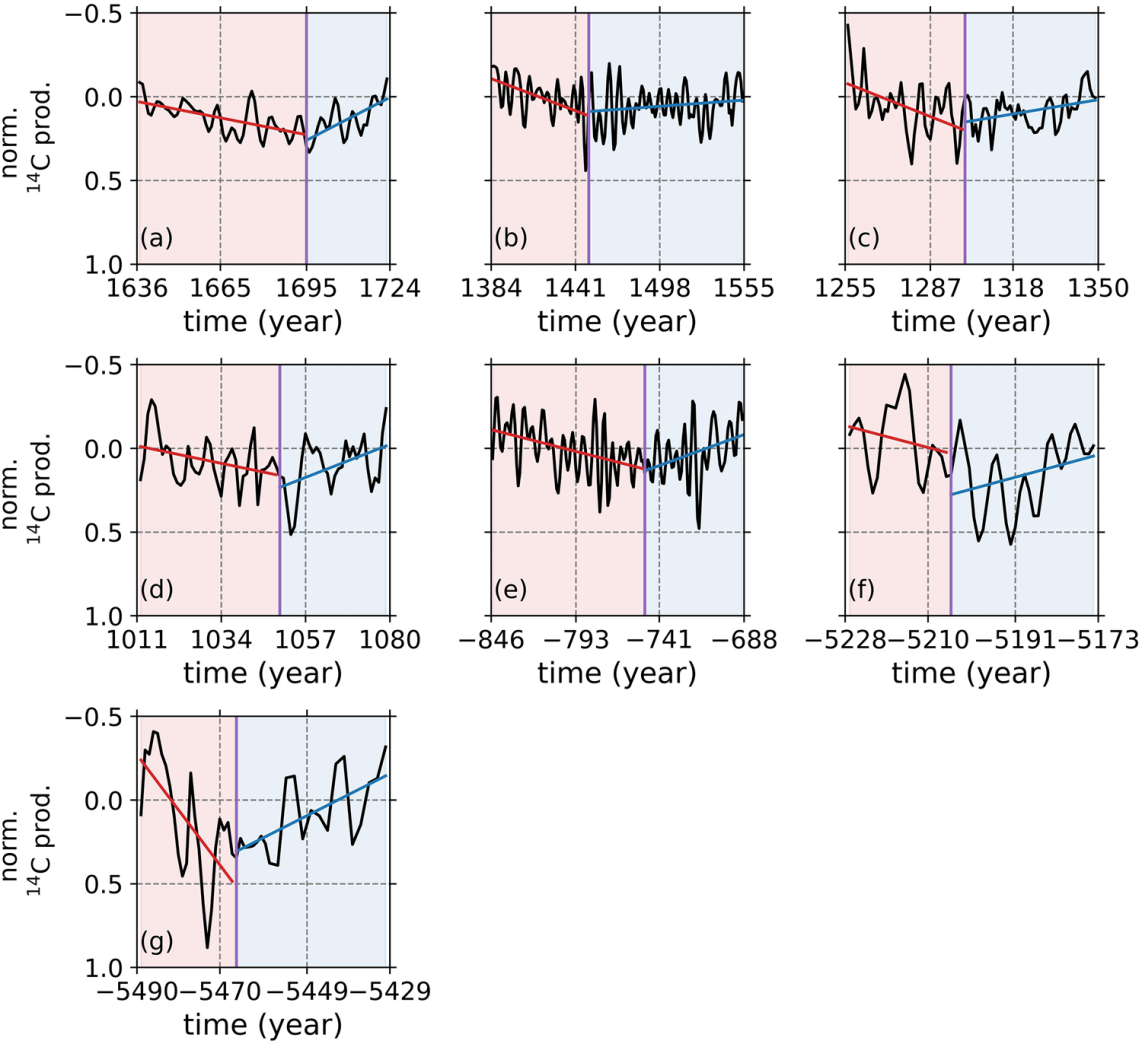
Each panel shows the Schwabe cycle dynamics during each 7 identified grand solar minima, where our annually-resolved compilation has full coverage. The red and blue linear fits show the change in the slope of solar activity levels on the onset and termination of the grand solar minima, respectively. The red and blue shades indicate the onset and termination of each grand solar minima. Note that the y axes are reversed.

To study the overall behavior of the solar dynamo during grand solar minima, we fitted linear models during the onset and termination of each minimum (Fig. 3). The durations of the onset and termination periods tend to show two types of variations: (i) a longer onset with shorter termination (Fig. 3a,d,e), and (ii) shorter onset with longer termination (Fig. 3b,c,f,g).

**Figure 4:**
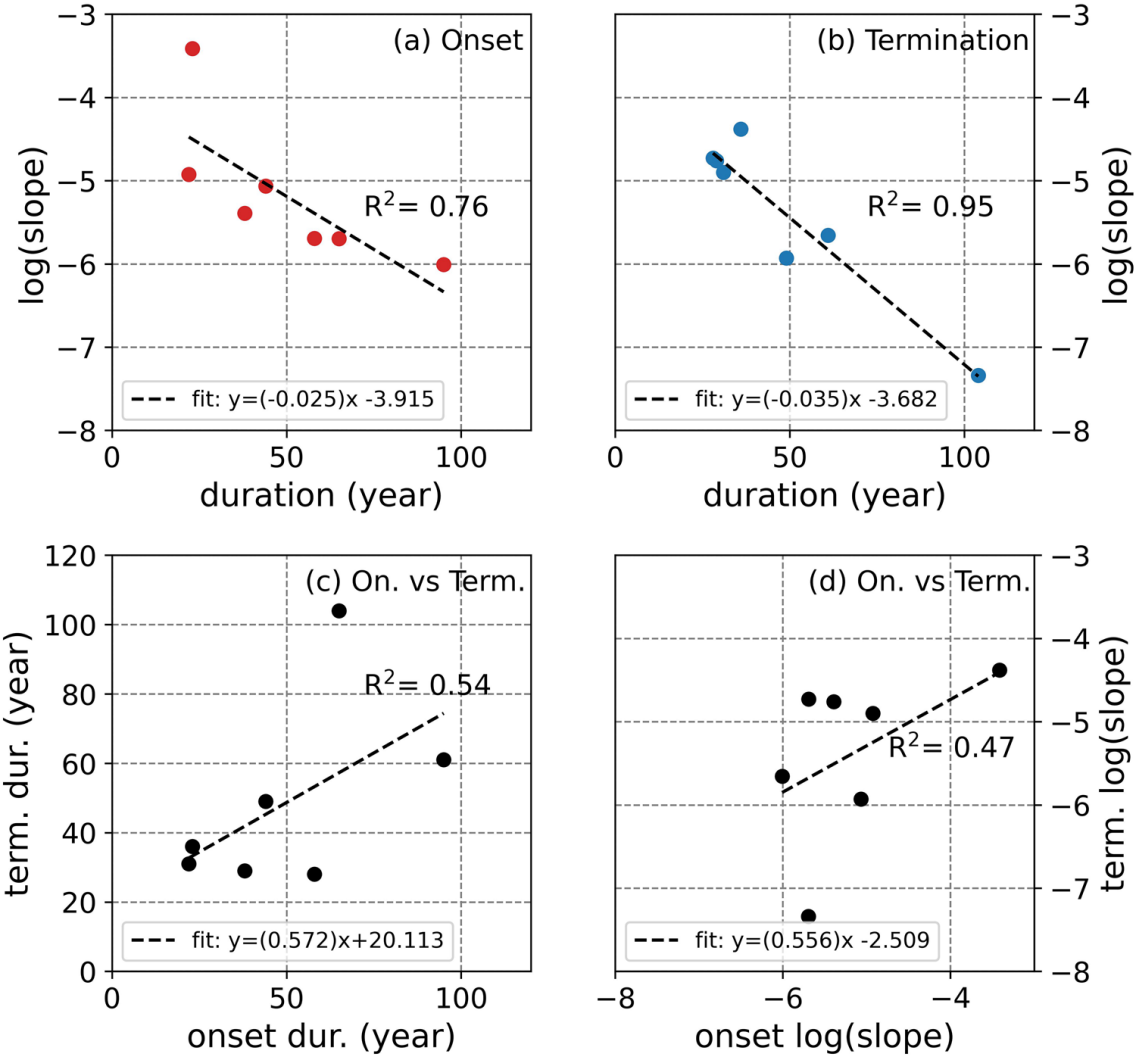
(**a**) Shows the relationship between the logarithm of the slope of solar activity levels and duration of each grand solar minimum onsets, while (**b**) shows the same for its termination. (**c**) Shows the relationship between the durations of the onsets and terminations of grand solar minima, while (**d**) shows the relationship between their slopes. Note that the slope values for terminations were inverted (multiplied by − 1) to calculate their logarithms.

We then investigated the relationship between how fast the Sun enters (exits) a grand minimum as determined by the slope of the linear fit during the onset (termination) of a grand solar minimum and its duration (Fig. 4). Our results show that there is a clear exponential relationship between the rate of decrease (increase) in solar activity levels and how long the onset (termination) of the grand solar minima will last (Fig. 4a,b, respectively). This means that if the decay or growth rate of solar activity level is known, the duration of the corresponding onset or termination period can be calculated. Another interesting result is that the relationships found for the onset and termination periods are very similar, which might indicate a global relationship. Additionally, we also investigated the relationship between the durations of onsets and terminations (Fig. 4c) and between the rate of increase and decrease of solar activity levels (Fig. 4d). These relationships show a weaker correlation around $R^{2}\sim \,0.5$ in comparison to $R^{2}=0.76$ and $R^{2}=0.95$ found for the relationships between the rate of decrease in solar activity levels and how long the onset of the grand solar minima will last, and increase in solar activity levels and how long the termination will last, respectively.

**Figure 5:**
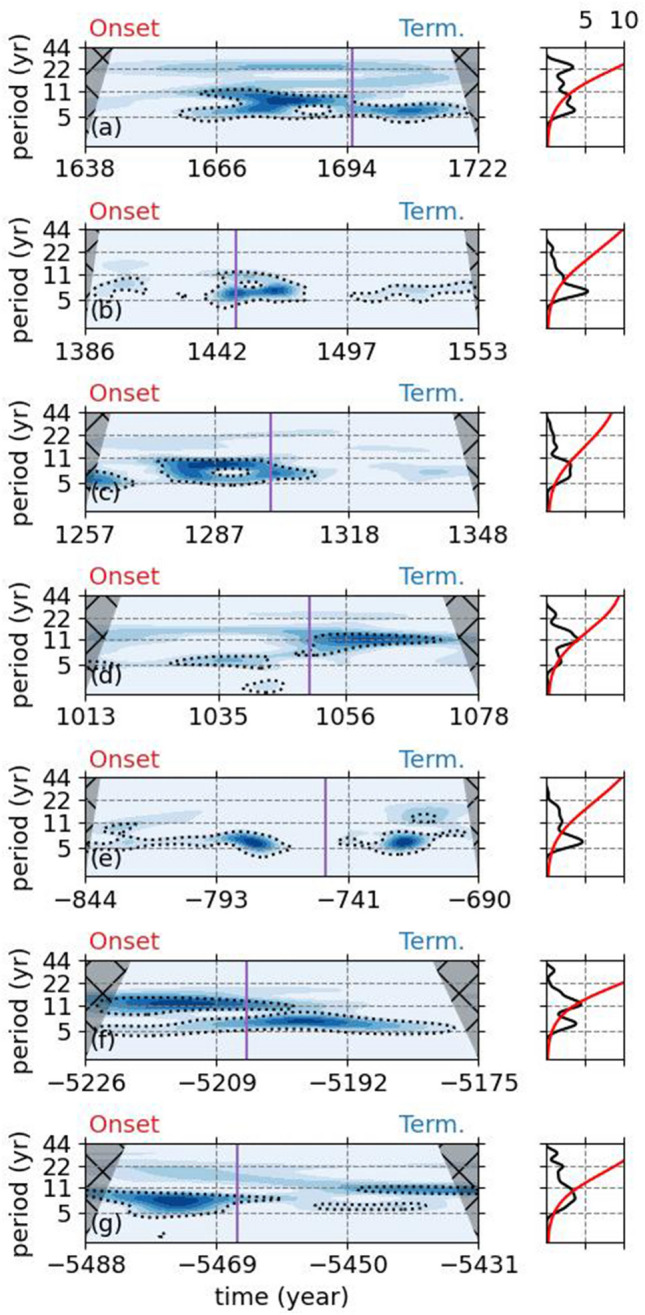
The CWTs and GWs of the high-passed ^14^C production rates with a cutoff frequency of (1/30) year^-1^ during grand solar minima. The black dotted lines in CWTs and red lines in the GWs display the statistical significance levels at a $\rho < 0.05$ level. The purple line shows the center of each minimum and taken from Refs.^10,12^.

To study the persistence of the Schwabe cycles during grand solar minima and variations in Schwabe cycle lengths and amplitudes, we once more high-passed filtered the data using a Butterworth filter of degree 5 with a cutoff frequency of 1/(30) year^-1^. This process allowed us to have a flat background for the Schwabe cycle amplitudes, independent of the longer term variations during each grand solar minimum. Following that we calculated the Continuous and Global Wavelet Spectrum (CWT and GW) for each 7 grand solar minima (Fig. 5) (see “Methods” section).

The behavior of the cycle lengths and amplitudes differ for different grand solar minimum. For example, during the Maunder Minimum (Fig. 5a), The CWT could not detect any signals which can be associated with the Schwabe cycles during early in its onset, while there are two signals with periods of $\sim$ 6 years and $\sim$ 11 years could be detected after around 1650s. Towards the end of the onset and beginning of the termination period, the $\sim$ 11 year periodicity becomes shorter and stays as $\sim$ 6 years until the end of the termination of the Maunder Minimum (Fig. 5a).

The Spörer Minimum, on the other hand, displays a different behavior (Fig. 5b). On the onset of the minimum, a low amplitude signal with a period of $\sim$ 8 years can be observed, which ceases to exist toward the end of the onset. Shortly before the onset and up until $\sim$ 1490, a period of $\sim$ 6 years with a higher amplitude becomes visible, the length of which becomes $\sim$ 8 years close to $\sim$ 1490. After a relatively short period of no signal, a periodicity of $\sim$ 6 years comes back after around $\sim$ 1497, which then becomes close to 11 years towards the end of the termination period of the Spörer Minimum (Fig. 5b). The Wolf Minimum exhibits a broad signal with periodicities of $\sim$ 6 years and $\sim$ 11 years with high amplitudes on its onset (Fig. 5c). Shortly after the end of onset and start of the termination period, these signals vanish. Even though there is a small amplitude signal can be observed with a period between 5 and 11 years, this signal is not statistically significant at 0.05 level (Fig. 5c). The Oort Minimum shows a statistically significant period of around 5 years during its onset, which then quickly becomes a high amplitude 11 year signal during its termination (Fig. 5d). The grand solar minimum between 844 BC and 690 BC displays an almost continuous signal with a period of around 6 years to 8 years, except for the short period between the end of its onset and its termination periods (Fig. 5e). The onset of the minimum between 5226 BC and 5175 BC shows a strong 11 year periodicity together with a smaller amplitude 5 year period (Fig. 5f). Following the end of its onset period, the 11 year period becomes 5 years with an increased amplitude, which persists until the end of the termination period of this minimum (Fig. 5f). An opposite behavior can be observed during the grand solar minimum between 5488 BC and 5431 BC (Fig. 5g). During the very early stages of its onset, there is a statistically significant periodicity of about 11 years, which then becomes shorter about 8 years with a high amplitude. This signal becomes statistically insignificant after the end of the onset during the termination period around 5445 BC, where a high amplitude 11-year signal, together with a weaker 6-year signal, comes back through the end of this grand minimum (Fig. 5g).

## Discussion

Our results clearly show that there is a clear exponential relationship between the rate in which the Sun goes into a grand minimum and the duration of its onset period. This relationship is even clearer when the Sun starts to recover from solar grand minima, as indicated by the relationship between the rate in which the solar activity levels increase and the duration of the termination period of grand solar minimum. Using these exponential relationships, we can predict the length of the duration of the onset (0.76) and the termination (0.95) periods by using the decay or growth rate of the solar activity levels. The governing relationships are also quite similar, which might indicate a more global relationship, rather than one for each period. The predictability of the durations of the onset and termination periods based on the rate of decrease and increase of solar activity levels, respectively, also suggests that a physical mechanism is responsible for these changes rather than random processes. The relationships between the duration of the onset and termination, and also between the rate of change in the solar activity levels during these two periods are, on the other hand, are weaker at a rate of $\sim$ 0.50.

Our results on the persistence and variations in Schwabe cycle lengths during grand solar minima are in line with previous studies^7,10,13,18–22,34^.

For example, peak to peak distances during the Maunder Minimum, which corresponds to our Fig. 5a, from the past production rates of ^14^C observed to start around 11 years, which then becomes around 6 years in the center of the minimum and then it recovers to be $\sim$ 11 years again toward the end of its termination^13^. Another study suggested that around 1650 the Schwabe cycle length become around 9 years, after which it recovers to being 11 years again^14^. However, it must be noted that the variability during this period is extremely high for this study. Using data from Danish Oak, it was also shown that after the center of the Maunder Minimum, the Schwabe cycles were around 7 years with an accompanying longer signal around 15 years, however only at a statistical significance level of 0.90^23^. Our results are partly in line with these results, except for the fact that our results do not show that the Schwabe cycles recover to be 11 years toward the end of the Maunder Minimum. For the Spörer Minimum, which corresponds to our Fig. 5b, peak to peak distances are shown to be longer than 11 years on its onset, which then becomes around 6 years toward the center of the minimum and recovers to be around 11 years again^13,14^. On the contrary, Ref.^34^ shows that the Scwabe cycles become gradually longer between 1370 and 1410 up to 16 years, while^23^ shows that the cycle length is around 4 years around 1470 which then becomes longer around 11 years toward its termination. Our results also indicate similar behavior, where the cycle lengths become around 6 years and it recovers to be around 11 years toward the end of the Spörer Minimum. As for the Wolf and Oort Minima, which corresponds to our Fig. 5c,d, it was observed that the cycle lengths starts at around 11-years and then they become shorter at around 6 years around the center of the minimum, and then they recover to be around 11 years again^13,14^. Our results also show similar behavior to these, except that we were not able to detect a statistically significant signal after around 1318. We must note that most of these studies rely on individual ^14^C records, while we draw our conclusions from a global compilation of all publicly available annually or bi-annually resolved ^14^C records.

Furthermore, the amplitude and length variations in the Schwabe cycles during grand solar minima are not the same for each grand solar minimum. Results from our high resolution ^14^C production rate compilation series suggests that there might be two or more different kinds of grand solar minima; one where the Schwabe cycle lengths decrease on the onset and stay the same during the termination phase of a grand minimum, and another one where the cycles lengths decrease on the onset of a grand minimum which then recovers to be around 11 year again.

Solar dynamo processes are expected to include random fluctuations in Reynolds stress, where angular momentum is transported among the plasma flows, as well as the emergence of magnetically strong confined regions^35–38^. However, despite these random fluctuations, the recurrent behavior of the Schwabe cycles with varying lengths and amplitudes, which also bears the memory from the previous cycles, are well known and documented^1,2,38,39^. The variations in the solar cycle lengths, as well as the amplitudes, are suggested to be linked to the meridional circulation in the Sun, which is a plasma flow that transports the magnetic flux from equatorial regions to the poles, after which it carries the magnetic flux below the base of the convection zone^40–43^. The shortening and lengthening of the Schwabe cycles as well as the variations in their amplitudes during grand solar minima can be explained by the variations in the meridional circulation rates and in the magnetic diffusivity levels and profiles throughout the solar convection zone^37,43,44^. For example, for lower magnetic diffusivity throughout the solar convection zone slower meridional circulation will result in stronger toroidal field as it will have enough time for the pre-existing poloidal field to built up. On the other hand, for higher magnetic diffusivities, slower meridional circulation will mean weaker toroidal field, as the pre-existing poloidal field will experience a faster diffusive decay^44^. In short, lower magnetic diffusivity will produce stronger Schwabe cycles with longer cycles, while the higher diffusivity will produce weaker cycles with longer cycles.

In conclusion, our results suggest that there might be two or more different types of grand solar minima, one of which shows shortening of the Schwabe cycles on the onset of the grand minimum, where it stays as is during its termination period. The second type is observed to display shorter than 11-year cycles on the onset and during the grand minima, which then recovers to be longer again during the termination period. These two types of behavior might suggest the magnetic diffusion profiles throughout the solar convection zone are affected differently during these grand minima. Using these results together with the shorter-term variations observed in the solar flow fields as constrains, we plan to run a comprehensive study using solar dynamos to investigate the possible physical mechanisms responsible for the grand solar minimum periods.

## Methods

### Box-diffusion carbon model for production rate calculations

To study the past variations in solar activity levels, we used annually and bi-annually resolved ^14^C measurements. Most of the data sets used in this study are available from the IntCal web page (https://www.intcal.org/). Additional data, some of which are not yet included in the IntCal database, can be found in Ref.^14^.

To calculate the past production rates of each ^14^C record, including the IntCal20 curve, we used a box-diffusion carbon model^45–48^. The box-diffusion model incorporates the CO_2_ exchange among the atmosphere, the biosphere, and upper ocean mixed layer and 42 deep-sea layers, where we kept the option for direct ventilation of the deep ocean off^33,46,47^. The model takes in CO_2_ and atmospheric ^14^C concentrations as inputs and outputs the ^14^C production rates, which are normalized to the modern day, pre-bomb, values.

Following^14^, to prepare the ^14^C concentration records, we first pad each annually and bi-annually resolved ^14^C record, from the left and the right of it, with the low resolution (LR) IntCal20 curve, linearly interpolate each record so they all have 0.1 year time resolution, and then apply a low-pass filter with a (1/6) year^-1^ cutoff frequency to remove any high-frequency variability in the data. The reason for this is to remove the known effects of the past sudden increases in ^14^C values, which are attributed to Coronal Mass Ejections (CMEs) and Solar Energetic Particle events (SEPs). Recently, it was shown that the influence of the Sun’s large scale dipolar magnetic field is stronger in variations in the GCR intensities through drift processes, and therefore production rates of cosmogenic radionuclides, for time scales longer than 5 years, while variations in shorter time scales stem from solar wind conditions, including large SPEs and CMEs, through diffusion and convection mechanisms^49^. Previous studies identified these types of short-term sudden increase in ^14^C production rates in high resolution ^14^C records, which are related to CMEs and SPEs, in 774–775^50^, 993–994^51^, and some smaller amplitude increases 1261–1262, 1268–1269, and 1279–1280 before the Wolf Minimum^52^. The durations of these events are generally shorter than 5 years and consistent with the findings of Ref.^49^. We must also note that the 774–775 AD, 993–994 AD, and 5410 BCE events^50,51,53^ are not among the time ranges that our study considers. The other events found in 1261–1262, 1268–1269, and 1279–1280 falls within the range between 1255 and 1350 (Fig. 3c), however these events are very weak, only $\sim$ 13%, $\sim$ 27%, and $\sim$ 19% of that of the 774–775 CE event^52^, and easily be eliminated by the applied filtering procedure.

The same pre-processing pipeline is also applied to the CO_2_ record, except for the padding process, which is created by splicing two records^54,55^ after inter-calibration. For each high resolution (HR) atmospheric ^14^C record. This procedure was repeated 10,000 times in an iterative process, where in each iteration the measurement errors for each ^14^C concentration record are added to the original signal as a Gaussian noise. Using the generated 10,000 ^14^C past production rates, we calculated the mean and standard deviation of the ^14^C production rates for each record.

The box-diffusion model does not incorporate the Suess effect^56^, which is defined as the reduction in the atmospheric ^14^C/^12^C ratios due to the increase in the anthropogenic CO_2_ emissions after 1850 AD. However, based on the available atmospheric CO_2_ records, we calculated the ^14^C production rates period up to 1950 AD. It must be noted that the ^14^C production rates after the termination of the Maunder Minimum in 1725 AD are not used.

### Compiling the high-resolution production rates

After calculating the past production rates of each ^14^C record under consideration, including that from the IntCal20, to create a global record of past production rates of the ^14^C, we compiled the HR ^14^C production rates using the Bootstrapping method. This method was previously used for compilations of cosmogenic radionuclide records^57^. Prior to compiling the records, we used a linear interpolation method to fill the gaps for bi-annually resolved ^14^C production rate records to obtain annual resolution, where necessary. The compilation method works as follows:

- each ^14^C production rate per year is re-sampled 10,000 times with added Gaussian white noise with an amplitude equal to its standard deviation, creating a distribution of potential values,
- if there is more than one ^14^C production rate for a particular year coming from different HR ^14^C production rate record, the first step is repeated for each measurement
- we then randomly choose 1000 values out of the distribution from the second step and take their average and create a distribution of 1000 means,
- as a final step, we calculate the mean and standard deviation of the distribution of means.

The compilation allowed us to have a global record of HR ^14^C production rates extending continuously back to 650 AD as well as 9 additional periods with gaps spanning a few centuries to millennia in between. Following the compilation of the HR ^14^C production rates, we linearly interpolate if there is a gap for only 1 year. The gaps more than 1 year are not interpolated.

In addition, even though there might be overlapping quasi-periodicities of $\sim$ 60, $\sim$ 200, and $\sim$ 2400 years in the geomagnetic field intensities and solar activity levels^30,33^, it was shown that the influence from the variations in geomagnetic field intensities on the ^14^C production rates for periods shorter than 500 years is relatively low and it only starts to be more effective on timescales between 300 and 500 years and longer^29–33^. We must note that in our study, the longest time scale we have is around 170 years, which is the duration of a grand solar minimum. Therefore, using a high-pass filter is effective in removing any possible influence of the variations in the geomagnetic field on the ^14^C production rates. Furthermore, the solar $\sim$ 2400 year cycle, the so-called Hallstatt cycle^58,59^, poses as a baseline in multi-millennial scale solar activity levels and the occurrences of grand solar minima and maxima are clustered around the Hallstatt cycle minima and maxima, respectively^60^. Therefore, it is shown to be very challenging to disentangle the effects of the variations in solar activity levels from those in geomagnetic field intensities on the ^14^C production rates for periods longer than 500 years^33^.

We need to point out that the main motivation of our study is to investigate the relative variations in lengths and amplitudes of the $\sim$ 11-year Schwabe cycles on the onset, during, and on the recovery of grand solar minima, maximum duration of which is found to be below 170 years^10,12^. Therefore, to remove the effects of the Hallstatt cycles, the high-frequency variations in solar activity from CMEs and SPEs, possible interference of the harmonics of the Schwabe cycles, and the effects from the geomagnetic field variations, we employed a high-pass filter to the data using a Butterworth filter of order 5 for the cutoff frequency of (1/250) year^-1^. Following investigating the solar activity dynamics during the solar grand minima, we once more applied a high-pass filter to the data using a Butterworth filter of order 5 for the cutoff frequency of (1/30) year^-1^ to study the variations in the Schwabe cycle lengths and amplitudes.

The Butterworth filter, which is maximally flat in the passband and does not cause any distortion in the low-frequency signal component^61^, is defined as;

1 $$\begin{aligned} \mid H_{B}(j\omega )\mid ^{2} = \frac{1}{1+(\omega / \omega _{c})^{2n}},\end{aligned}$$

where $\omega _{c}$ and *n* denote the cutoff frequency and the order of the Butterworth filter. To filter the data we used *SciPy package*^62^ in python.

To filter the HR ^14^C compilation, we padded the data with the LR ^14^C compilation for sections where there are large discontinuities in the HR ^14^C compilation.

### Continuous and global wavelet transformations

To investigate the possible periodicities in the LR and SR ^14^C production rates during grand solar minima, we use Continuous and Global Wavelet Transformations (CWT and GW) using *pycwt* library in python. The data sets are first standardized using their individual mean and standard deviation values before each wavelet analyses.

The dominant modes of variability in a time series and their evolution in time are detected using the CWTs by decomposing them^63^. A wavelet is defined as a function with zero mean that is localized in frequency and time domains^64^. There are various wavelet functions, such as Mexican Hat, Meyer, but the most commonly used wavelet function is the Morlet wavelet, which is a complex symmetric function. The Morlet wavelet is defined as

2 $$\begin{aligned} \psi _{0} (\eta ) = \pi ^{-1/4} e^{i \omega _{0} \eta } e^{-\eta ^{2}/2}, \end{aligned}$$

where $\omega _{0}$ and $\eta$ are the dimensionless frequency and time^63^. A balance between time and frequency localisation can be reached using $\omega _{0} = 6$, which also allows detection of peaks and valleys^64,65^. The convolution of time series ($x_n$) with the normalized and scaled wavelet, which is defined as the CWTs of a time series with uniform time increments of $\delta t$, is given by

3 $$\begin{aligned} W^{X}_{n} (s) = \sqrt{\delta t/s}\sum _{n' = 1}^{N} x_{n'}\psi _{0} \left[ \left( n' - n \right) \delta t/s \right] , \end{aligned}$$

where *s* is scale. The wavelet power is then calculated as $|W^{X}_{n} (s)|^2$, where the complex argument of $W^{X}_{n} (s)$ is the local phase^63,64^.

The Global Wavelet transformations (GWs), on the other hand, are calculated by averaging over all the local wavelet spectra in time and is given as^63^;

4 $$\begin{aligned} {\overline{W}}^{2}_{n} (s) = \frac{1}{N}\sum _{n = 0}^{N-1}\mid W_{n} (s) \mid ^{2}. \end{aligned}$$

The GWs give an estimate for the background variation in the data together with their consistent and unbiased estimation of the true power spectrum^63^.

The statistical significances of the CWT and GWs are calculated using Monte Carlo method based on the each time series first order auto-regressive correlation coefficients (AR1). In our study, we used 500 ensembles of surrogate data set pairs with the same AR1 coefficients as the input data sets. For some periods, it was not possible to calculate the AR1 coefficient due to numerical reasons. For these periods, we used white noise surrogates.

## Data Availability

The datasets used and/or analysed during the current study are available from the corresponding author on reasonable request.
