# Author Correction: Exploring solar dynamo behavior using an annually resolved carbon-14 compilation during multiple grand solar minima

**DOI:** 10.1038/s41598-024-57261-1

**Published:** 2024-03-21

**Authors:** Fadil Inceoglu

**Affiliations:** 1 Cooperative Institute for Research in Environmental Sciences, University of Colorado Boulder, Boulder, CO 80309 USA; 2 National Centers for Environmental Information, National Oceanic and Atmospheric Administration, Boulder, CO 80309 USA

Correction to: *Scientific Reports* 10.1038/s41598-024-55317-w, published online 07 March 2024

The original version of this Article contained repeated errors. As a result in the Abstract,

“Our results from 7 grand solar minima showed a clear relationship between the rate of increase (decrease) in solar activity levels and how long the onset (termination) of the grand solar minima will last.”

now reads:

“Our results from 7 grand solar minima showed a clear relationship between the rate of decrease (increase) in solar activity levels and how long the onset (termination) of the grand solar minima will last.”

In the Results,

“Our results show that there is a clear exponential relationship between the rate of increase (decrease) in solar activity levels and how long the onset (termination) of the grand solar minima will last (Fig. 4a,b, respectively). This means that if the growth or decay rate of solar activity level is known, the duration of the corresponding onset or termination period can be calculated.

These relationships show a weaker correlation around R2 ∼ 0.5 in comparison to R2 = 0.76 and R2 = 0.95 found for the relationships between the rate of increase in solar activity levels and how long the onset of the grand solar minima will last, and decrease in solar activity levels and how long the termination will last, respectively.”

now reads:

“Our results show that there is a clear exponential relationship between the rate of decrease (increase) in solar activity levels and how long the onset (termination) of the grand solar minima will last (Fig. 4a,b, respectively). This means that if the decay or growth rate of solar activity level is known, the duration of the corresponding onset or termination period can be calculated.

These relationships show a weaker correlation around R2 ∼ 0.5 in comparison to R2 = 0.76 and R2 = 0.95 found for the relationships between the rate of decrease in solar activity levels and how long the onset of the grand solar minima will last, and increase in solar activity levels and how long the termination will last, respectively.”

In the Discussion,

“Using these exponential relationships, we can predict the length of the duration of the onset (0.76) and the termination (0.95) periods by using the growth or decay rate of the solar activity levels.

The predictability of the durations of the onset and termination periods based on the rate of increase and decrease of solar activity levels, respectively, also suggests that a physical mechanism is responsible for these changes rather than random processes.”

now reads:

“Using these exponential relationships, we can predict the length of the duration of the onset (0.76) and the termination (0.95) periods by using the decay or growth rate of the solar activity levels.

The predictability of the durations of the onset and termination periods based on the rate of decrease and increase of solar activity levels, respectively, also suggests that a physical mechanism is responsible for these changes rather than random processes.”

The original Article has been corrected.

